# Authors’ Reply: Why Medical Education Without Artificial Intelligence Still Matters: A Neuroscience-Informed Perspective

**DOI:** 10.2196/95205

**Published:** 2026-03-31

**Authors:** Juan S Izquierdo-Condoy, Marlon Arias-Intriago, Laura Montero Corrales, Esteban Ortiz-Prado

**Affiliations:** 1 One Health Research Group, Universidad de Las Américas, Via Nayon S/N, Quito, Pichincha, 170124, Ecuador, 593 0992561230; 2 Escuela de Comunicación, Latin University of Costa Rica, San José, Costa Rica

**Keywords:** artificial intelligence, medical education, machine learning, adaptive learning systems, future implications

 We thank the author of the pertinent commentary, *“*Why Medical Education Without Artificial Intelligence Still Matters: A Neuroscience-Informed Perspective” [1]. We believe this letter makes a valuable contribution to the debate on artificial intelligence (AI) in medical education by emphasizing the cognitive consequences of training clinicians in environments where these tools are available yet not always reliable or accessible in everyday practice.

From our perspective, the author’s argument expands on a concern we had already raised previously: if AI, particularly generative AI, can compromise critical thinking and cognitive autonomy, this erosion may also translate into a reduced capacity to sustain clinical reasoning when the tool fails or is unavailable [2]. In our article, “Artificial Intelligence in Medical Education: Transformative Potential, Current Applications, and Future Implications” [3], we identified uncritical dependence on algorithmic outputs as one of the main limitations of incorporating AI into medical training.

The neurocognitive evidence cited by the author reinforces this argument. Recent studies suggest that the use of generative AI may alter how individuals engage in complex tasks. Although this evidence remains preliminary, it is consistent with what has already been described regarding automation bias and cognitive off-loading. Likewise, accepting automated suggestions without sufficient analysis may increase the risk of error when the system fails or is unavailable [4,5]. Taken together, these findings support a central idea: AI should support reasoning, not replace expert judgment.

In our viewpoint, we addressed these concerns through concrete proposals. Importantly, our discussion was not limited to generative AI but also encompassed other educationally relevant technologies, including natural language processing, intelligent tutoring systems, virtual reality, and augmented reality. In this context, we advocated for AI literacy curricula that would enable students not only to use these tools but also to critically evaluate them, recognize their limitations, identify biases, and assess their outputs against sound clinical reasoning. We also supported governance structures aligned with international ethical frameworks, such as the UNESCO (United Nations Educational, Scientific and Cultural Organization) Recommendation on the Ethics of Artificial Intelligence and the World Health Organization guidance on the ethics and governance of AI in health [3]. We consider human oversight, transparency, bias monitoring, and accountability to be essential safeguards for the responsible integration of AI.

We also agree with the author’s suggestion to include structured exercises in which students must function without AI support, as this may strengthen independent diagnostic reasoning and decision-making under uncertainty. At the same time, this discussion must acknowledge persistent technological inequalities, particularly in low-income countries, where access to advanced AI tools remains uneven [6].

We agree that AI integration in medical education should not displace human intelligence but rather enhance professional competence without undermining clinical autonomy or critical thinking. Preparing future physicians to function appropriately with or without AI will be essential to protect patient safety, respond across diverse contexts, and preserve the human dimension of medicine. We again thank the author for this valuable contribution and concur on the need for further empirical evidence, particularly longitudinal studies, to guide a prudent, ethical, and evidence-based integration of AI into medical education.

## Abbreviations

AI: artificial intelligence
UNESCO: United Nations Educational, Scientific and Cultural Organization
